# Early Experience with High-density Electroanatomical Mapping Using the Rhythmia™ Mapping System in Congenital and Pediatric Heart Disease

**DOI:** 10.19102/icrm.2021.120901

**Published:** 2021-09-15

**Authors:** Joshua M. Saef, Brendan J. Burke, Patrick J. Tchou, Peter F. Aziz

**Affiliations:** ^1^Department of Cardiology, Cleveland Clinic Foundation, Cleveland, OH, USA; ^2^Department of Pediatrics, Cleveland Clinic Children’s, Cleveland, OH, USA; ^3^Department of Pediatric Cardiology, Cleveland Clinic Children’s, Cleveland, OH, USA

**Keywords:** Adult congenital heart disease, congenital heart disease, electroanatomic mapping, fluoroscopy, Rhythmia

## Abstract

The Rhythmia™ system (Boston Scientific, Natick, MA, USA) facilitates the rapid acquisition of high-resolution electroanatomical and activation maps. However, there are limited data on its efficacy and safety in pediatric and adult congenital heart disease (CHD) patients. In a retrospective, observational cohort study, adult CHD and pediatric patients followed by pediatric cardiology underwent electrophysiologic study using the Rhythmia™ electroanatomic mapping system. Variables examined included the number of electroanatomical maps required, acquisition time, procedure time, fluoroscopy time, radiation dosage, and rate of recurrent arrhythmia. Twelve consecutive patients, including six male patients (50%), were included with an average age of 27.7 years (range: 11–64 years). Seven (58%) of these patients had a diagnosis of CHD [moderate complexity in two (17%) and great complexity in five patients (42%)] and 10 (83%) patients underwent ablation. A total of 37 high-density maps were created in 12 procedures, with a median of 8,140 mapping points, taking a median of 631 seconds. The median procedure time was 189.5 minutes. The median fluoroscopy time was 0.9 minutes, with eight (67%) patients receiving no fluoroscopy at all. Recurrence occurred in one patient (8%) over a median follow-up duration of 16 months (interquartile range: 12.8–17.3 months). No adverse periprocedural events were recorded. This study suggests the use of high-density electroanatomic mapping in adult CHD patients showed potential for rapid acquisition of highly detailed maps with minimal fluoroscopy time or risk of periprocedural events in the studied population.

## Introduction

Innovation in the medical and surgical treatment of congenital and pediatric cardiovascular anomalies has led to a marked improvement in outcomes, with almost 85% of patients surviving into adulthood.^1^ As the congenital heart disease (CHD) population has grown, cardiac dysrhythmias have proven to be a concerning source of morbidity.^2,3^ The heterogeneity of inherent cardiac structural anomalies and procedural history within the CHD population has resulted in substantial anatomic variability and a wide spectrum of dysrhythmia mechanisms.^4,5^ Catheter ablation in the CHD population is an accepted therapeutic modality, and accurate mapping of the relevant electrophysiological substrate is crucial for its success.^6,7^

The Rhythmia™ electroanatomic mapping system (Boston Scientific, Natick, MA, USA) is a recent advance in catheter technology capable of rapid and high-resolution electroanatomic and activation mapping performed intraprocedurally with minimal manual annotation, whereas alternate systems require extensive manual reannotation.^8,9^ Its adoption is being explored in the catheter ablation of atrial and ventricular arrhythmias in adult populations, with studies indicating a potential to decrease fluoroscopy time, the use of contrast agents, and procedure time.^10^ The system has been trialed in pediatric patients with dysrhythmia and adult patients with CHD, albeit only in small studies and case reports, making it difficult to decipher its efficacy.^11–13^

Given the sparseness of available data and difficulty applying the experience of other operators to a diverse population in clinical decision-making, further defining the safety and accuracy of the Rhythmia™ mapping system in this population is warranted. The potential for decreased radiation exposure and an improvement in mapping accuracy may be meaningful in CHD patients, who are often younger and suffer lower rates of acute and long-term success for ablation compared to patients with traditional cardiac anatomy.^4,14,15^

## Methods

### Study design

We performed a retrospective, observational cohort study. Patients with a CHD diagnosis or being followed by pediatric electrophysiology with a history of sustained arrhythmia that had been studied using the Rhythmia™ electroanatomic mapping system at the Cleveland Clinic were considered for analysis. Study participant selection favored those in whom there was a concern for complex and/or multiple flutter circuits or anatomical constraints that could limit the ability to map. The complexity of CHD was categorized according to the Anatomic and Physiological classification system developed by the writing committee of the 2018 American Heart Association/American College of Cardiology Guideline for the Management of Adults with Congenital Heart Disease. Data were collected for patients who were studied between January 1, 2018, and December 31, 2019. This study was approved by the Cleveland Clinic Foundation Institutional Review Board.

### High-resolution mapping acquisition

All patients signed an informed consent form for the relevant procedure. Vascular access was obtained under ultrasound guidance. Diagnostic catheters were positioned to serve as timing references. The INTELLAMAP Orion™ mapping catheter (Boston Scientific) was placed through an 8-French (Fr) sheath, which is similar to comparable mapping and ablation catheters. Chamber mapping was conducted with minimal or no fluoroscopy prior to diagnostic catheter placement, as described by Nagaraju et al.^16^ Heparin was administered as deemed necessary by the operator to achieve therapeutic anticoagulation. The Orion™ 64-pole basket mapping catheter was advanced into the systemic venous cardiac chambers and vena cavae unless the patient had a Fontan palliation. If a left or pulmonary venous origin was suspected, transseptal punctures were performed under echocardiography with or without fluoroscopic guidance. Programmed extrastimuli, isoproterenol, and/or intravenous (IV) calcium were used to induce the clinical arrhythmia. Maps were considered complete when chamber anatomy was reconstructed with the best electrode–tissue contact achievable as determined by the operator, using the Orion™ multi-electrode catheter. If arrhythmia was inducible, each substrate was mapped until a full cycle length (CL) was achieved. Entrainment maneuvers were used to prove participation of atrial myocardium prior to ablation. If necessary, additional anatomic mapping was performed with the ablation catheter. Phrenic nerve testing was performed before ablation in the posterior right atrium (RA) when appropriate.

### Ablative intervention

The INTELLATIP MIFI™ open-irrigated catheter (Boston Scientific) was used for ablation during procedures. Radiofrequency ablation (RFA) was performed at a maximum power of 30 to 40 W. After ablation procedures were completed, repeat programmed extrastimuli, isoproterenol, and/or IV calcium were used to provoke arrhythmias until myocardial refractoriness or a 200-ms CL was achieved. Bidirectional block was confirmed in cases where RFA was used to disrupt macro-reentrant circuits. Acute procedural success was defined as noninducibility of the targeted clinical arrhythmia and/or achievement of bidirectional block.

### Follow-up

Patients were followed up with at outpatient clinical visits one to six months after their index procedure. When present, pacemakers and implantable cardioverter-defibrillators were assessed through remote monitoring, while ambulatory monitoring was conducted in patients without implantable devices. Twelve-lead electrocardiograms were obtained at outpatient office visits. Recurrence was defined as any arrhythmia episode lasting 30 seconds or longer noted on implantable device monitoring or Holter electrocardiogram. If there was a concern for recurrence based on clinical symptoms or history, additional outpatient clinical visits and continuous monitoring were offered.

### Offline analysis

Maps of the clinical arrhythmias were reviewed and cataloged offline using the Rhythmia™ software. Data stored included the volume mapped (calculated automatically by the system), areas of dense scar (summation of all distinct areas of voltage < 0.05 mV) low voltage (0.05–0.5 mV), and characteristics and timing of electrograms for use in Boston Scientific’s Lumipoint™ algorithms.

### Statistical analysis

Continuous variables are expressed as median values with the first and third quartiles [interquartile range (IQR)]. If clinically relevant, minimum and maximum values are also indicated. Comparative statistics were established using the Mann–Whitney U nonparametric test. Results were considered statistically significant if the p-value was less than 0.05.

## Results

### Patients

We evaluated 12 consecutive pediatric or CHD patients who underwent an electrophysiologic study with the Rhythmia™ mapping system [average age: 27.7 years (range: 11–64 years)]. Six (50%) participants were male. Seven (58%) patients had a diagnosis of CHD, and 10 (83%) patients underwent ablative procedures. CHD conditions consisted of moderate complexity in two patients (17%) and great complexity in five patients (42%). Patient characteristics are outlined in **Table 1**.

### Arrhythmias

Aside from the two patients with premature ventricular contractions, all other patients’ clinical arrhythmias were supraventricular in origin. Amongst CHD patients, surgical scar from prior baffling, atriotomy, and shunts were often found to contain the site of earliest activation. A total of 16 arrhythmias were treated with ablation in 10 patients. Arrhythmias could not be induced in four cases, and substrate mapping was only performed in sinus rhythm.

Atrial tachyarrhythmias were mapped and/or treated within the RA in most (six of eight) cases, though scar within a Fontan conduit and a Mustard baffle were also culprit sites. The median CL of mapped atrial tachyarrhythmias was 350 ms (IQR: 288.3–441 ms). In three of five cases, atrial flutter was typical with a cavotricuspid isthmus–dependent circuit. The median CL of mapped atrial flutters was 275 ms (IQR: 251.5–275.5 ms).

A median of 14 (IQR: 8–27) RFA applications were applied per arrhythmia treated. Acute procedural success, defined as noninducibility by pacing or isoproterenol, was achieved in all patients who underwent ablation **(Table 2)**.

### Procedural characteristics: congenital heart disease and pediatric patient groups

Individual demographics, structural cardiac diagnoses, prior surgeries, catheterization interventions, ablation history, clinical arrhythmias, medical therapy, procedural characteristics, and ablation targets for included patients with and without CHD are detailed in **Tables 3 and 4**, respectively. There were no statistically significant differences between these groups with regard to procedure time [190 (135–298) vs. 189 (383–320) minutes; p = 0.749], fluoroscopy time [10.3 (0–11.4) vs. 0 (0–1.9) minutes; p = 0.682], or air kerma [0.02 (0–0.03) vs. 0 (0–0.01) mGy; p = 0.682].

### Procedural characteristics: fluoroscopy and radiation

The median fluoroscopy time of our cohort was 0.95 minutes, with eight (67%) of our patients receiving no fluoroscopy at all. The median air kerma was 0.005 mGy (IQR: 0–0.03 mGy).

### Procedural characteristics: structural congenital heart disease considerations

#### Tetralogy of Fallot

An atrial tachycardia was targeted in one patient with previously repaired tetralogy of Fallot (patient 1). The Orion™ catheter was used to map the inferior vena cava (IVC), RA, and superior vena cava (SVC) with an FJ bidirectional decapolar catheter (Biosense Webster, Diamond Bar, CA, USA) in the coronary sinus and a quadripolar catheter in the right ventricular apex. RA burst pacing, programmed extrastimuli, high-dose isoproterenol, and IV calcium were unable to induce sustained atrial tachycardia. As such, mapping was only performed in normal sinus rhythm.

#### Fontan palliation

A total of three arrhythmias were treated in two patients with prior Fontan palliation, though clinical arrhythmia was only induced in one patient.

**Figure 1:**
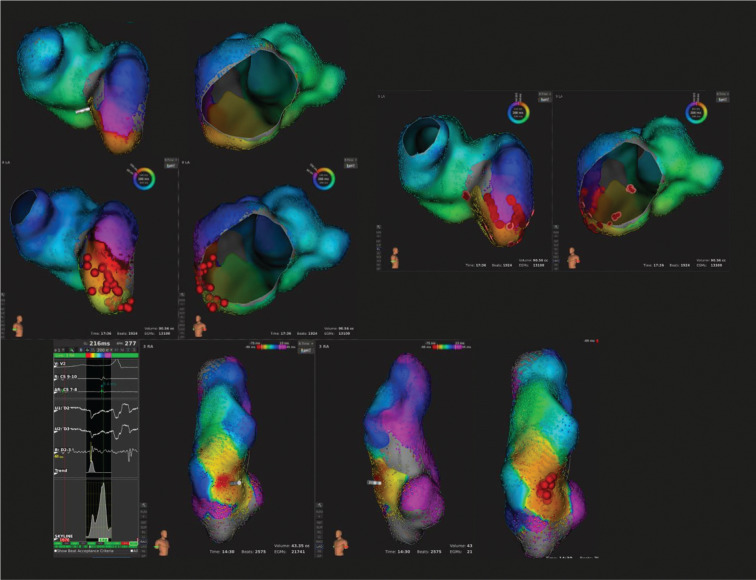
The case of a patient (patient 2) with hypoplastic left heart syndrome post–lateral Fontan. Images show electrograms, isochronic activation mapping (red indicating the earliest activation and purple indicating the latest scaled in milliseconds), and ablation sites (red points) of a tricuspid isthmus–dependent atrial flutter within the RA [upper six images, right anterior oblique (RAO) and left anterior oblique (LAO) projections] and a focal, automatic tachycardia in the lateral Fontan (bottom three images, RAO and LAO projections). The electrogram ablation catheter tracing signal is well before the atrial and the coronary sinus tracings, and the unipolar catheter tracing shows a Q-wave, all suggesting placement at the origin of the atrial tachycardia.

In the case where arrhythmia was induced (patient 2), two arrhythmias were treated. A focal atrial tachycardia (CL ~250 ms) was mapped with earliest local activation at the anterolateral aspect of the Fontan and was easily ablated. Subsequent atrial burst pacing induced an atrial flutter (CL ~280 ms) that was mapped within the Fontan to be anterior and inferior to a plugged fenestration. Ablation at the site did not terminate the tachycardia. As such, transbaffle access was obtained into the RA using a Mullins sheath (Medtronic, Minneapolis, MN, USA) and a BRK needle (Abbott, Chicago, IL, USA) with electrocautery. RA mapping showed a tricuspid isthmus-dependent atrial flutter (counterclockwise), and the MIFI™ catheter was used to create an RFA line from the tricuspid annulus to the baffle, which terminated the atrial flutter, as seen in **Figure 1**. Bidirectional block was confirmed with sinus activation and voltage mapping.

**Figure 2:**
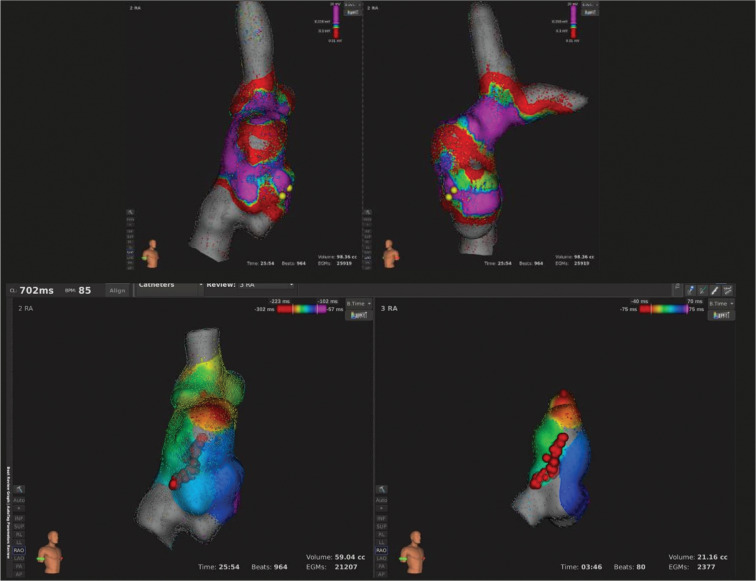
The case of a patient (patient 3) with hypoplastic left heart syndrome post–fenestrated Fontan who developed an atrial flutter. The voltage maps [top two images, RAO and LAO projections] of the SVC, RA, IVC, and left pulmonary artery seen here show a scar below the sinus node and low-voltage area down to the IVC at the anterolateral RA. The isochronic activation maps (bottom two images) show the RA in an RAO projection in sinus rhythm after treatment, with no wave progression through ablation lesions (red points). The scale for the voltage maps is from 0.01 (red) to 20 mV (purple). The isochronic activation maps indicate the earliest activation with red color and the latest activation with purple color in milliseconds.

In the second case (patient 3), ultra–high-density mapping was performed in sinus rhythm given the inability to induce clinical arrhythmia, as seen in **Video 1**. An empiric RFA lesion was created from an anterolateral Fontan scar to the superior border of the IVC. After the ablation, the Orion™ catheter was placed back in the Fontan circuit and sinus activation showed a clear block across the RFA lesion, as seen in **Figure 2 and Video 2**.

#### D-transposition of the great arteries with atrial baffling

Two patients with prior atrial-switch procedures for D-transposition of the great arteries were studied and treated, including one with symptomatic premature atrial contractions (PACs) with paroxysms of sustained supraventricular tachycardia (SVT) and the other with multiple SVTs leading to hospitalization. Both had a prior history of atrioventricular (AV) nodal reentrant tachycardia ablation. The patient with PACs also had undergone a prior ablation for atrial tachycardia within the systemic venous baffle.

**Figure 3:**
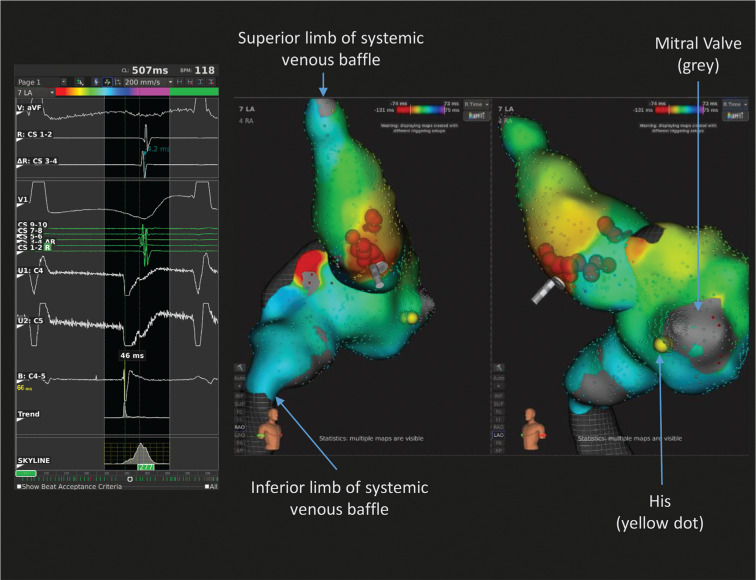
The case of a patient (patient 4) with D-transposition of the great vessels post–Mustard procedure. Image shows electrograms, isochronic activation mapping, and ablation lesions (red points) within the IVC, SVC, and systemic venous Mustard baffle from the RAO and LAO projections. An atrial tachycardia with earliest local activation at the anterosuperior aspect of the baffle was ablated. The electrogram shows earliest activation in the coronary sinus leads. A Q-wave is seen in the unipolar lead, suggesting that this is the site of exit for the focal atrial tachycardia. The isochronic activation maps indicate the earliest activation with red color and the latest activation with purple color in milliseconds.

In the case of symptomatic PACs (patient 4), the Orion™ catheter and a decapolar catheter were placed in the systemic venous baffle. There was initial difficulty advancing the Orion™ catheter into the SVC baffle, so it was exchanged for the MIFI™ ablation catheter to create a matrix, which was used to find a pathway to advance the Orion™ catheter. An activation map of the PAC showed earliest local activation at the anterosuperior aspect of the baffle. RFA delivered at the site immediately suppressed PACs, as seen in **Figure 3**.

**Figure 4:**
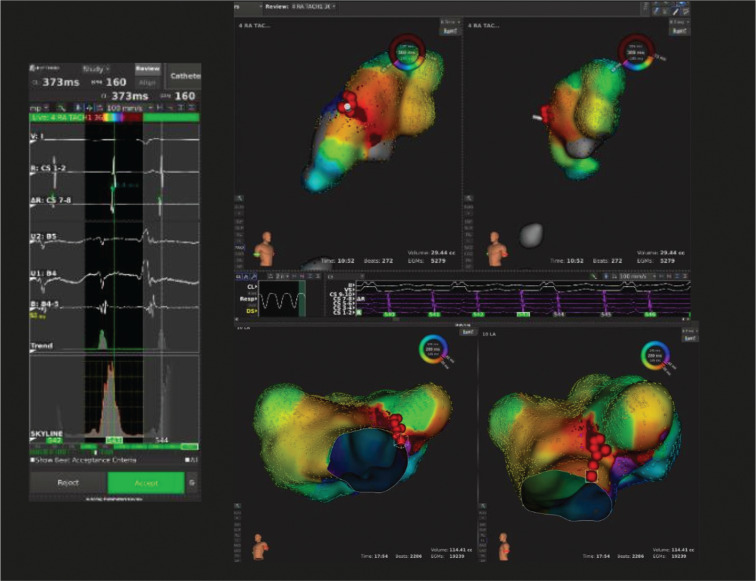
The case of a patient (patient 5) with D-transposition of the great vessels post–Mustard procedure. Mapped territory consists of the IVC, systemic venous baffle, left (systemic venous) atrium, and right (pulmonary venous) atria. The top two images show isochronic activation maps and ablation of an atrial tachycardia with the region of earliest activation on the superior margin of the patient’s systemic venous baffle. The electrogram shows a signal close to but not at this ablation site. The bottom two images show mapping and ablation (red points) of a subsequently induced atrial flutter in the pulmonary venous atrium. The isochronic activation maps indicate the earliest activation with red and the latest activation with purple in milliseconds.

The other patient (patient 5) presented to the electrophysiology lab with refractory SVT (CL ~450 ms). The Orion™ catheter initially was used to map the IVC, systemic venous baffle, left (systemic venous) atrium, and mitral valve annulus. A region of earliest activation was mapped on the superior margin of the baffle, and ablation terminated the tachycardia, as seen in **Figure 4**. Moments later, a faster narrow complex tachycardia spontaneously initiated with a different activation pattern (CL ~390 ms). The systemic venous portions of the heart were mapped again, but the regions of earliest activation appeared more widespread in the region of the baffle. Transbaffle access was pursued using a small curve Agilis sheath (Abbott) with a transseptal needle, and the Orion™ catheter was advanced into the pulmonary venous atrium. Mapping showed areas of activation 30 and 40 ms pre–P-wave from the ridge just outside of the appendage. The area was ablated but this did not alter the tachycardia; a second activation map showed a region of early-meets-late wavefront just outside of the ridge, and an RFA ablation line extending from a region of scar superior to the ridge all the way down to the ridge’s inferior margin terminated the tachycardia. Repeat programmed extrastimulation induced an even faster narrow complex tachycardia with an atrial CL of approximately 290 ms. A new activation map of the left-sided pulmonary venous atrium showed an atrial flutter rotating in a clockwise fashion and the left posteroinferior atrium in the pulmonary venous atrium, and a lesion was attempted through this corridor. As we approached the tricuspid valve annulus, the patient’s AV conduction slowed. Given the concern for inducing heart block, the operator decided not to proceed with further ablation of this (likely nonclinical) tachycardia.

#### Patients with multiple congenital heart disease diagnoses

A pediatric patient (patient 6) with situs inversus, an AV canal defect, total anomalous pulmonary venous return, malposed great arteries, pulmonary atresia, and major aortopulmonary collateral arteries requiring multiple prior surgical repairs was assessed in the context of recurrent, symptomatic atrial flutter requiring multiple cardioversions. The Orion™ catheter was introduced into the systemic RA. An Inquiry catheter (Abbott) was placed in the esophagus as a reference catheter. An anatomic shell and voltage map were created in sinus rhythm, showing extensive scarring, especially at the anterior and septal aspects of the right atrium. Atrial burst pacing, programmed extrastimulation, and isoproterenol infusion were unable to induce the tachyarrhythmia.

An adult patient (patient 7) with previously repaired patent ductus arteriosus, aortic coarctation, and pulmonary stenosis with atrial flutter (CL ~250) was studied due to medication intolerance and breakthrough. Voltage mapping during coronary sinus pacing revealed a lateral scar consistent with his atriotomy scar, with areas of low-amplitude fractionated signals inferiorly. There was also a posteroseptal scar with areas of continuous low-amplitude signaling. A horizontal RFA line was created from the posteroseptal scar to the atriotomy scar, and then another vertical RFA line was established down to the IVC.

### Follow-up

Over a median of 16 months (IQR: 12.8–17.3 months), only one patient in the cohort experienced recurrence of their clinical arrhythmia or the need for repeat electrophysiology study. Two patients (17%) were able to discontinue all antiarrhythmic therapy. No patients experienced acute complications or late postprocedural adverse events.

The patient who experienced recurrence had an inherited two *SCN5A* pathogenic variants causing sinus node dysfunction and atrial standstill with an extensive history of atrial flutter and atrial tachycardia (patient 10). Her tachycardia was unable to be induced during the study, and a cavotricuspid RFA line was placed empirically. She experienced an episode of syncope and collapse with two minutes of unconsciousness. Device interrogation at the time of the event showed two episodes of very high ventricular rates from 245 to 265 bpm, both lasting less than one minute. She underwent placement of an atrial lead to prevent bradycardia-induced atrial arrhythmias, to provide antitachycardia pacing, and for diagnostic purposes.

## Discussion

We report our initial experience with the Rhythmia™ mapping system in pediatric patients and patients with CHD. Our approach in this cohort was unique in that we mapped the target chamber with the Orion™ catheter with minimal or no fluoroscopy and without internal reference catheters. In our initial experience, we found this technique to be both fast and effective while minimizing fluoroscopy and procedure times.

A total of 37 high-density maps were created, with a median of 8,140 mapping points within a median of 631 seconds. Our median procedure time in this cohort was 189.5 minutes, which is comparable to previous experience with this technology^17,18^ [Mantziari et al.: 285 (IQR: 194–403) minutes; Ernst et al.: 184 (IQR: 155–298) minutes]. Of note, provider unfamiliarity with the software and hardware likely contributed to increased procedure times and will likely improve with increased familiarity. No manual annotation was required during map creation, which is consistent with previous reports.^8,12,17^ No internal reference catheters were required in this experience as the Rhythmia™ mapping system allowed for the external surface electrode to serve as a reference instead.

We report a high success rate and minimal symptomatic arrhythmia recurrence over a median follow-up period of 16 months. In addition, two patients were able to de-escalate off all antiarrhythmic medications. Our patient selection being based on complex and/or multiple flutter circuits or anatomical constraints that could limit the ability to map precluded a straightforward comparison to a propensity-matched control. It should be noted that our outcomes were significantly better compared to similar cohorts^18^; however, our small sample size and differing techniques limit the significance of this finding.

Our one documented episode of symptomatic recurrence occurred in a patient with *SCN5A* channelopathy. *SCN5A* pathogenic variations are thought to have variable expressivity and can cause variable phenotypes, including Brugada syndrome, long QT syndrome, and cardiac conduction disease, depending on the type of mutation and patient factors.^19–21^ *SCN5A* channelopathies are unlikely to respond to endocardial ablation in the long term and often require epicardial intervention or other alternative therapies.^22,23^

The Rhythmia™ mapping system requires induction of a sustained arrhythmia in order to create an accurate activation sequence map. The most apparent advantage of the Rhythmia™ mapping system with the Orion™ catheter is the rapid acquisition of activation, which is imperative in cases where the arrhythmia of interest is difficult to induce or sustain, or in patients with CHD due to heterogeneous anatomy and scar/fibrosis. In cases where arrhythmias could not be induced, high-resolution maps were obtained in sinus rhythm, used to identify scar, and empiric scar modification was performed (patients 3 and 7). If areas of the targeting arrhythmia were still inducible, defining the precise location of anatomical “breakthrough” was facilitated by electroanatomic remapping, which is often rapid and easy to define. Given the high number of electroanatomic points generated rapidly during mapping, both the clinical operator and the Rhythmia™ representative constantly checked the electrical signals to ensure consistency, removing or modifying points as needed.

Catheter visualization is occasionally a challenge due to external impedance reference, with the mapping catheter occasionally displayed as moving erratically when actually still, even completely, disappearing from the software display at times. In discussion with Boston Scientific, this was thought to be a result of inconsistent impedance through the chest wall and is an area for improvement for future iterations of this technology. With these current difficulties with catheter localization with external impedance references, we recommend utilizing internal impedance reference catheters until these issues are resolved.

There is a significant learning curve with the Orion™ catheter and the Rhythmia™ mapping system due to differences in steering and pressure indication when compared to other mapping systems, with a detrimental effect on the ease of use. The recent addition of the INTELLANAV MIFI™ XP Ablation Catheter (Boston Scientific) claims to improve upon both of these issues.^24^

The use of fluoroscopy in our patient cohort was mostly to facilitate transseptal or transbaffle puncture. Our technique of minimal or no fluoroscopy results in significantly less fluoroscopy time than similar cohorts.^17,18^ Adult CHD patients are exposed to a significant cumulative effective dose of radiation.^25^ With increased radiation exposure due to diagnostic and therapeutic interventions, adult CHD is associated with an increased prevalence of cancer.^25,26^ Even low-dose ionizing radiation was found to be independently associated with an increased odds of cancer in adult CHD patients.^27^ This is of particular concern as the frequency of these procedures is increasing and starting to be performed in progressively younger patients, thus increasing the cumulative radiation exposure and risk for cancer.^28^ This technology in combination with intracardiac echocardiography (ICE) presents a promising path to reducing procedural radiation exposure in adult CHD patients associated with RFA.

Boston Scientific requires universal use of anticoagulation with the Orion™ basket catheter, with a suggested goal activated clotting time of more than 300 seconds.^29^ The main drawback of universal anticoagulation is the theoretical increase in hematoma risk at vascular access points, although this was not seen in our cohort. The necessity of heparin in all cases with Orion™ catheters complicates the decision to place additional catheters midprocedure, and practitioners should be cognizant of the potential need for additional catheters. It is our institutional practice to use ultrasound guidance for all arterial access in our electrophysiology labs. Ultrasound guidance was used in all procedures, which has been shown to significantly improve procedural success and reduce complications.^30^ In cases that would benefit from the Orion™ catheter, there is an argument to start select cases with arterial access with a 4-Fr catheter to allow for upsizing if needed midprocedure.

Our cohort includes a large proportion of great complexity CHD cases and a similar to larger proportion of great-complexity CHD cases than seen in previous cohorts.^17,18^ Ernst et al.^18^ conducted a similar case series; however, they avoided procedures requiring transbaffle or retrograde access due to concerns for increased procedure time and fluoroscopy time. We elected to perform transbaffle mapping and ablations in two of our patients due to arrhythmia severity and given our methodology of mapping without fluoroscopy and the use of ICE. We found that transbaffle mapping was feasible and, in complex CHD, easily facilitated with ICE imaging. We again advocate minimal fluoroscopy in addition to utilization of ICE for all our congenital patients as able to avoid unnecessary procedural radiation and to assist in defining anatomy.

We reported no procedural complications in our cohort, including in patients with complex CHD.

### Future directions

This initial report shows promising efficacy; however, larger prospective trials are needed to determine the efficacy and safety of this product.

### Limitations

This retrospective cohort study reflects a preliminary, single-center experience using the Rhythmia™ mapping system in an adult CHD population. We had high rates of success in a variety of adult CHD cases, but our findings are limited by the small number of enrolled patients. Operator unfamiliarity with the Orion™ catheter and Rhythmia™ mapping software may have influenced the procedure times. The cohort presented is heterogeneous in their anatomy and presenting arrhythmia, which limits the ability to generalize these findings.

## Conclusions

This preliminary cohort study of the use of the Rhythmia™ high-density mapping system in adult CHD patients demonstrates the potential for rapid acquisition of highly detailed maps and quick conversion to scar modification, with minimal procedure and fluoroscopy times. Our technique mapping without fluoroscopy was not only fast and effective but significantly decreased fluoroscopy time in combination with ICE. Due to catheter display errors in the current implementation of Rhythmia™ with an external impedance reference, we recommend internal impedance reference catheters at this time.

## Figures and Tables

**Video 1:** High-density electroanatomic activation map of Fontan in normal sinus rhythm prior to empiric ablation.

**Video 2:** High-density electroanatomic map of Fontan in normal sinus rhythm. Given the history of atrial flutter, an empiric ablation line was made between baffle scarring. Bidirectional block is demonstrated postprocedurally.

**Table 1:** Patient Characteristics

Characteristics	
Baseline parameters (mean ± standard deviation)	
Age, years	28 ± 16.4
Male sex, n (%)	6 (50%)
Height, cm	164.9 ± 13.9
Weight, kg	69.4 ± 23.2
Body mass index, kg/m^2^	25.1 ± 7.3
Arrhythmia indicating study, n (%)	
Atrial tachycardia	8 (67%)
Atrial flutter	5 (42%)
Supraventricular tachycardia not otherwise specified	1 (8%)
Premature atrial contractions	1 (8%)
Premature ventricular contractions	2 (17%)
Prior ablation	4 (33%)
CHD, n (%)	7 (58%)
Moderate complexity	2 (17%)
Great complexity	5 (42%)
Medication, n (%)	
Class I antiarrhythmic	1 (8%)
Class II antiarrhythmic	3 (25%)
Class III antiarrhythmic	6 (50%)
Class IV antiarrhythmic	2 (17%)
Anticoagulation	7 (58%)
Device, n (%)	
Permanent pacemaker	2 (17%)
Implantable defibrillator	2 (17%)

**Table 2:** Procedural Characteristics and Outcomes

Procedural Parameters	
Number of arrhythmias ablated, n (%)	
0	2 (17%)
1	7 (58%)
2	3 (25%)
Anatomic structure mapped, n (%)	
Atrium	7 (58%)
Atrial baffle	2 (17%)
Fontan	2 (17%)
Right ventricle	2 (17%)
Chamber volume, mL (IQR)	
Atria	125 (115–140.5)
Atrial baffle	66.5 (59.3–73.8)
Fontan	51.5 (47.8–55.3)
Arrhythmia cycle length, ms (IQR)	
Atrial tachycardia	350 (288.3–441)
Atrial flutter	275 (251.5–275.5)
Intracardiac echocardiography, n (%)	4 (33%)
Intraprocedural transesophageal echocardiography, n (%)	1 (8%)
Fluoroscopic time, min (IQR)	0.9 (0–11.4)
Air kerma, mGy (IQR)	0.005 (0–0.03)
Procedure time, min (IQR)	189.5 (149.5–323.75)
RFA applications per arrhythmia (IQR)	14 (8–27)
Mapping time, s (IQR)	631 (455–977)
Mapping points (IQR)	8,140 (4,993–11,905)
Procedural outcomes, n (%)	
Recurrent arrhythmia	1 (8%)
Discontinued antiarrhythmic drugs	2 (17%)

IQR: interquartile range.

**Table 3:** Congenital Heart Lesions, Prior Surgeries, Catheterization Interventions, Ablation History, Arrhythmia, and Ablation Target

Patient Number	Age, years	CHD Diagnos(es)	ACHD AP Classification	Prior Surgical Treatment(s)	Prior Catheter Treatment(s)	Prior Ablation Treatment(s)	Arrhythmia(s) Indicating Study	Antiarrhythmic Drug(s)	Anticoagulation	Device	Fluoro Time, min	Air Kerma, Gy	Intraprocedural Imaging	Anatomic Arrhythmia Target(s)	Ablation
1	43	Tetralogy of Fallot	IIb	Right-sided Blalock–Taussig shunt Complete repair	Percutaneous valve placement in the left and right pulmonary arteries (failed attempt at percutaneous pulmonary valve replacement due to large annulus)	Atypical atrial flutter ablation (RA free wall)	Atrial tachycardia	Metoprolol	None	None	0	0	None	Unable to induce	None
2	24	Hypoplastic left heart syndrome Atrial septal defect	IIIc	Norwood operation Bidirectional cavopulmonary anastomosis Fenestrated Fontan	Percutaneous closure of Fontan fenestration Vascular plug in a baffle leak		Atrial tachycardia Atrial flutter	Sotalol	Warfarin	Epicardial DC-ICD	11.3	0.03	ICE	Anterolateral aspect of Fontan Cavotricuspid isthmus	Targets ablated
3	23	Hypoplastic left heart syndrome Bilateral SVCs without bridging vein Anomalous origin of the left coronary artery from the right pulmonary artery	IIId	Norwood operation Repair of anomalous left coronary artery Bilateral bidirectional Glenn Lateral tunnel fenestrated Fontan Tricuspid valve repair	Rashkind atrial septostomy Fontan fenestration closure		Atrial flutter	Sotalol Verapamil	Warfarin		0	0	None	Unable to induce	Empiric RFA lesion from the anterolateral Fontan scar to the superior border of the IVC
4	35	D-transposition of the great vessels	IIIb	Mustard procedure	Rashkind atrial septostomy SVC stenting	AV nodal slow pathway modification Atrial tachycardia ablation within the systemic venous baffle	Premature atrial contractions	Sotalol	Apixaban	DC-PPM	0	0	None	Anterosuperior aspect of the SVC/systemic venous baffle	Target ablated
5	48	D-transposition of the great vessels Congenital subpulmonic and pulmonic obstruction	IIIc	Mustard procedure Glenn procedure		AVNRT ablation	Atrial tachycardia Atrial flutter	Nadolol Amiodarone	Apixaban		15.4	0.06	ICE	Superior margin of the systemic venous baffle Pulmonary venous atrial appendage Isthmus between the left inferior pulmonary vein and the tricuspid annulus	Targets ablated
6	11	Situs inversus Common atrioventricular septal defect Total anomalous pulmonary venous return Malposed great arteries Pulmonary atresia Major aortopulmonary collateral arteries s/p conduit stenting	IIIc	Repair of total anomalous pulmonary venous return Construction of the common ventricle to pulmonary artery conduit Atrial septation Plication of right ventricular aneurysm Reconstruction of the ventricle to pulmonary artery conduit	Bilateral pulmonary artery stenting SVC stenting Ventricle to pulmonary artery conduit stenting		SVT	Sotalol Digoxin	Warfarin	Single-chamber, atrial PPM (implanted during the Rhythmia™ procedure)	10.3	0.02	TEE	Unable to induce	None
7	64	Coarctation of the aorta Patent ductus arteriosus Congenital pulmonic stenosis	IIb	Patent ductus arteriosus ligation Pulmonic stenosis repair with subvalvular resection Coarctation repair with subclavian flap Recoarctation repair with ascending to descending aortic graft	Transcatheter pulmonary valve replacement		Atrial flutter	Mexiletine Carvedilol Sotalol Digoxin	Warfarin	DC-ICD	11.5	0.03	None	Right atrial scar from prior atriotomy and posteroseptal scar	Horizontal RFA line from the posteroseptal scar to the atriotomy scar, then another vertical RFA line down to the IVC
											10.3 (IQR: 0–11.4)	0.02 (IQR: 0–0.03)			

ACHD: adults with congenital heart disease; AP: anatomic and physiological; AV: atrioventricular; CHD: congenital heart disease; DC-PPM: dual-chamber permanent pacemaker; DC-ICD: dual-chamber implantable cardioverter-defibrillator; ICE: intracardiac echocardiography; IQR: interquartile range; IVC: inferior vena cava; RA: right atrium; s/p: status post-; SVC: superior vena cava; SVT: supraventricular tachycardia; TEE: transesophageal echocardiography.

**Table 4:** Patients Without Structural CHD

Patient Number	Age, years	Cardiac Diagnos(es)	Prior Ablation Treatment(s)	Arrhythmia(s) Indicating Study	Antiarrhythmic Drugs	Anticoagulation	Device	Fluoro Time, min	Air Kerma, Gy	Intraprocedural Imaging	Anatomic Arrhythmia Target(s)	Ablation
8	20	Tachycardia-mediated cardiomyopathy	None	Atrial tachycardia	None	None	None	0	0	None	Mid-posterior wall of the right atrium	Target ablated
9	17	Familial-dilated cardiomyopathy	None	Atrial flutter Atrial tachycardia	None	None	None	1.9	0.01	ICE	Cavotricuspid isthmus Posterior right atrium along the border of the crista terminalis	Target ablated Target ablated
10	16	Sinus node dysfunction Atrial standstill Inherited *SCN5A* mutation	None	Atrial flutter	None	Warfarin	Single-chamber, ventricular PPM	43	0.1	ICE	Unable to induce	Empiric RFA line to the cavotricuspid isthmus
11	19	PVC-mediated cardiomyopathy	Failed PVC ablation	PVCs	Verapamil	None	None	0	0	None	Basal inferolateral RV near the tricuspid valve	Target ablated
12	12	Dilated cardiomyopathy	None	PVCs	None	None	None	0	0	None	Posteroseptal aspect of RVOT	Target ablated
								0 (IQR: 0–1.9)	0 (IQR: 0–0.01)			

ICE: intracardiac echocardiography; IQR, interquartile range; PPM: permanent pacemaker; PVC: premature ventricular contractions; RFA: radiofrequency ablation; RV, right ventricle; RVOT, right ventricular outflow tract.
